# Response to Letter to Editor: Headache After Sealing of Cerebrospinal Fluid Leaks in Patients With Spontaneous Intracranial Hypotension

**DOI:** 10.1111/ene.70452

**Published:** 2026-04-17

**Authors:** Adrian Scutelnic, Nedelina Slavova, Eric Morel, Franz Riederer, Tomas Dobrocky, Eike I. Piechowiak, Ralph T. Schär, Christoph J. Schankin

**Affiliations:** ^1^ Department of Neurology Inselspital, Bern University Hospital, University of Bern Bern Switzerland; ^2^ Institute of Diagnostic and Interventional Neuroradiology, Inselspital, Bern University Hospital, University of Bern Bern Switzerland; ^3^ Department of Neurosurgery Inselspital, Bern University Hospital, University of Bern Bern Switzerland; ^4^ Department of Neurology, Centre for Migraine and Headache Bellevue Medical Group Zurich Switzerland

We thank Fu et al. for their comments on our paper. We agree that multivariable logistic regression analyses might provide insights into predictors of persistent headache in our patient population. However, due to the heterogeneity of the study population with, for example, multiple leak types and the overall low number of patients, we decided against such analyses. Such a study would have been underpowered with its inherent limitations. Also, due to the rarity of this patient population, we instead chose to provide descriptive data only to avoid type I and II errors provided by fortuitous significant differences. The number of patients with new or recurrent CSF leak was also too low (*n* = 2) to form an adequate group for statistical comparisons.

We aimed to explore the clinical characteristics of a special patient population that might pose substantial difficulties in clinical routine: patients with persistent headache despite successful sealing of a CSF leak. In our opinion, invasively treated SIH patients without persistent headache are the best control group since this is the only group with the same diagnosis and intervention, which might allow meaningful comparisons.

We thank the authors for raising the issue of randomization of treatment. Given the rarity of SIH, a randomized control trial on treatment would require a large‐scale collaboration between specialized centers. Based on the findings of our paper, such a study would indeed be the next step.

## Conflicts of Interest

The authors declare no conflicts of interest.

